# Inactivation of the *sfgtr4* Gene of *Shigella flexneri* Induces Biofilm Formation and Affects Bacterial Pathogenicity

**DOI:** 10.3390/microorganisms8060841

**Published:** 2020-06-04

**Authors:** Abdelmoughit Kaoukab-Raji, Latéfa Biskri, Abdelmounaaïm Allaoui

**Affiliations:** 1 Laboratoire de Bactériologie Moléculaire, Faculté de Médecine, Université Libre de Bruxelles, Route de Lennik, 808, 1070 Brussels, Belgium; abdelmoughit.kaoukab@gmail.com (A.K.-R.); latefa.biskri@um6p.ma (L.B.); 2 Laboratoire de Microbiologie Moléculaire, CIPEM (Centre de Coalition, Innovation, et de prévention des Epidémies au Maroc), Mohammed VI Polytechnic University (UM6P), Lot 660, Hay Moulay Rachid, 41130 Benguérir, Morocco; 3 Medical Application Interface Center (CIAM), Mohammed VI Polytechnic University (UM6P), 43150 Benguérir, Morocco; 4 Microbiome and African Genome Center (AGC), Agrobiosciences department Mohammed VI Polytechnic University (UM6P), Lot 660, Hay Moulay Rachid, 41130 Benguérir, Morocco

**Keywords:** *Shigella* pathogenicity, glycosyltransferase, biofilm formation, cell invasion, lysozyme degradation, T3 secretion, PMNs killing

## Abstract

Biofilm formation is a significant cause for the environmental persistence of foodborne pathogens. This phenomenon remains misunderstood in *Shigella* *flexneri* whose pathogenicity is mainly associated with the virulence plasmid pWR100. Sequence analysis of the latter predicts a putative lipopolysaccharides (LPS) glycosyltransferase (Gtr) encoded by *Sfgtr4*, which is the second gene of the *SfpgdA-orf186-virK-msbB2* locus. We demonstrated here that purified SfGtr4 exhibited a Gtr activity in vitro by transferring glucose to lipid A. To establish the role of SfGtr4 in virulence, we generated a *Sfgtr4* mutant and assessed its phenotype in vitro. *Sfgtr4* mutant significantly reduced HeLa cells invasion without impairing type III effectors secretion, increased susceptibility to lysozyme degradation, and enhanced bacterial killing by polymorphonuclear neutrophils (PMNs). SfGtr4 is related to proteins required in biofilm formation. We established conditions whereby wild-type *Shigella* formed biofilm and revealed that its appearance was accelerated by the *Sfgtr4* mutant. Additional phenotypical analysis revealed that single *SfpdgA* and double *SfpgdA*-*Sfgtr4* mutants behaved similarly to *Sfgtr4* mutant. Furthermore, a molecular interaction between SfGtr4 and SfPgdA was identified. In summary, the dual contribution of SfGtr4 and SfPgdA to the pathogenicity and the regulation biofilm formation by *S. flexneri* was demonstrated here.

## 1. Introduction

*Shigella flexneri* is a Gram-negative pathogenic bacterium that causes human bacillary dysentery. Its ability to enter epithelial cells is conferred by a ~214-kb pWR100 plasmid encoding a Type 3 secretion apparatus (T3SA) [1]. A designated entry region of 30-kb of this plasmid encodes the Mxi and Spa proteins known to be involved in T3SA assembly, including the: (i) translocators IpaB, IpaC, and IpaD; (ii) effectors (IcsB, IpgB1, IpgD, and IpaA); (iii) chaperones (IpgA, IpgC, IpgE, and Spa15); and (vi) transcription activators VirB and MxiE (reviewed in [2]). Plasmid pWR100 also contains several genes encoding proteins required for bacterial cell-to-cell spread, such as IcsA [2]. Despite the significant progress made to unravel the content of pWR100, to date, several genes remain uncharacterized. In the present work, we investigated the function of *orf186*, the second gene located in the *SfpgdA-orf186-virK-msbB2* locus. The first three genes share 93% DNA sequence identity with *shf, capU*, and *virK* of *Escherichia coli* (EAEA 042) [3].

The equivalent of SfPgdA in Gram negative bacteria includes *E. coli*’s PgaB and *Yersinia pestis*’ HmsF, both of which are required in biofilm development [4,5]. The lipoprotein PgaB is known to introduce a limited amount of glucosamine into the poly-β-1,6-*N*-acetyl-D-glucosamine (PGA) polymer and therefore facilitate its export via the porin PgaA [5]. HmsF possesses a polysaccharide deacetylase activity [4] and contains the COG1649 domain, required for biofilm development [6]. Up to date, the closest well-characterized counterpart to both SfPgdA and Shf is the IcaB protein found in *Staphylococcus epidermidis,* which is also known to be involved in biofilm formation [7]. IcaB is a secreted protein involved in the mechanism of intercellular adhesion but also acts as a poly-N-AcGlc deacetylase of the exopolysaccharide (EPS) [8]. 

It is well known that planktonic cells produce EPS in response to a variety of environmental signals such as magnesium deficit and subsequently facilitating bacterial attachment [9]. Optimal expression of the *SfpgdA-orf186-virK-msbB2* locus is associated with low Mg^2+^-containing media and is regulated by the PhoP/PhoQ two-regulatory component system [10,11]. In a previous study, we reported that *Shigella* strains lacking either PhoP or SfPgdA are highly susceptible to lysozyme degradation and are less resistant to killing by polymorphonuclear neutrophils (PMNs) [12].

Sequence alignment revealed that *orf186* of *S. flexneri,* encodes a predicted LPS glycosyltransferase 4 (Gtr4). Orf186, renamed here SfGtr4 (for *S. flexneri* Gtr), shares 73% sequence identity with RfbU of *E. coli* [13]. RfbU is also involved in LPS biosynthesis and contains a RfaG domain associated with biofilm formation [14]. Gtrs catalyze monosaccharides transfer from an activated donor, such as a sugar-nucleotide, to an acceptor molecule [15]. Gtrs may therefore transfer UDP-, ADP-, GDP-, or CMP-sugar from activated donor molecules to specific acceptor molecules (e.g., lipid, protein, heterocyclic compound, or carbohydrate residue), reflecting a wider range of biological functions (CAZy database, http://www.cazy.org/GT1_bacteria.html).

Here, we sought to determine whether biofilm formation occurred in *Shigella* and to establish if Sfgtr4 is involved in such process. We found that biofilm formation was increased by the deletion of *Sfgtr4* gene, and phenotypical analysis revealed that Sfgtr4 and SfpdgA are both required to fulfill *Shigella* infection of HeLa cells or PMNs.

## 2. Material and Methods

### 2.1. Bacterial Strains and Growth Conditions

Strains used in this study are derivative from the wild-type *Shigella flexneri* 5 strain M90T-Sm [16] (Table 1). Bacteria were grown in tryptic casein soy broth (TSB, Becton Dickinson, Belgium) at 37 °C. *E. coli* strain DH5α λpir was used for the propagation of plasmids carrying an *oriT* origin of replication (pSW23T) [17], and SM10 λpir was used to transfer derivatives of pSW23T to *S. flexneri. E. coli* Top10 (Invitrogen, Carlsbad, CA, USA) was used for recombinant proteins production. *E. coli* strains were grown in Luria–Bertani (LB) medium (Becton Dickinson, Belgium) supplemented with appropriate antibiotics: ampicillin, 100 µg mL^−1^; kanamycin, 50 µg mL^−1^; streptomycin, 100 µg mL^−1^ and chloramphenicol, 25 µg mL^−1^ (VWR, France).

### 2.2. Construction of Single sfgtr4 and Double sfpgdA-sfgtr4 Mutants

Upstream and downstream regions of *SfpgdA* and *Sfgtr4* genes were PCR-amplified from plasmid pWR100. Plasmids and primers used in this study are listed in Table 1 and Table 2. Primers contain restriction sites used for DNA cloning. The amplified DNA fragments, containing a *Bam*HI site at the 5′ end and a *Sal*I site at the 3’, were subsequently cloned into the corresponding sites of the pTZ18R vector to generate pKA2 and pKA85 plasmids. pKA2 was used to generate plasmid pKA5 by inserting the 850-bp *Sma*I DNA fragment carrying the *aphA-3* (Km^R^) gene [20] into the unique *Eco*RV site of pKA2. Plasmid pKA85 was used to generate pKA120 by replacing the internal *Nru*I-*Eco*RV DNA fragment of pKA85 by *SmaI* digested *aphA*-3 gene. *SfpgdA* was interrupted at codon 120 while *Sfgtr4* was interrupted at codon 53. Next, an inactivated copy in each construct was inserted into the suicide vector pSW23T to generate pKA8 (*Sfgtr4::aphA-3*) and pKA121 (*SfpgdA-Sfgtr4::aphA-3*). The resulting plasmids were next transferred to *S. flexneri* M90T-Sm by conjugal mating and the structures of the two *Sfgtr4* and *SfpgdA-Sfgtr4* mutants were further confirmed by PCR. Plasmids pKA2 (SfGtr4), pKA1 (SfPgdA) [12], and pKA85 (SfPgdA and SfGtr4) were used in the complementation experiments.

### 2.3. Proteins Secretion and HeLa Cells Invasion

Crude extracts and concentrated culture supernatants of *S. flexneri* strains were prepared as previously described [12,16,21]. Proteins were separated on SDS-PAGE stained with Coomassie blue or analyzed by Western blot using anti-IpaB and anti-IpaC monoclonal antibodies (mAbs) [22,23]. Virulence of the various strains was evaluated by testing bacterial ability to invade HeLa cells. The gentamicin protection assay, used to assess bacterial invasion of HeLa cells, was carried out as previously described [23,24]. Briefly, 2 mL of *Shigella* strains were grown to exponential growth phase ([OD600nm] = 0.4) and centrifuged onto plates containing 2 × 10^5^ HeLa cells at 2000 *g* for 10 min. After 1 h of incubation at 37 °C, cells were washed three times with 2 mL EBSS-earle’s balanced salt solution (Gibco, Belgium) and further incubated for 1 h in 2 mL MEM–minimum essential media (Gibco, Belgium) containing gentamicin at 50 µg mL^−1^ final concentration. The plates were washed three times and incubated for 15 min in Triton X-100 (0.1 % in PBS) to lyse the cells. Bacterial solutions were then diluted and plated on TSB-Agar containing appropriate antibiotics for colony-forming unit (CFU) counting.

### 2.4. Lysozyme Sensitivity Assay and Polymorphonuclear Neutrophils (PMNs) Bacterial Infection

The ability of *Sfgtr4* mutant to resist lysosomal degradation was assessed as described previously [12]. Briefly, the pellet of bacteria grown to exponential phase was washed with Phosphate-Buffered Saline (PBS) (Gibco, Belgium) and then incubated for 4 h at 37 °C with 40 µg mL^−1^ of lysozyme and 0.1 mg mL^−1^ of lactoferrin (Sigma-Aldrich, Overijse, Belgium) before CFU counting. PMNs were isolated according to Pycock’s method [25]. Bacterial infection by PMNs was performed as we described previously [12]. In brief, bacterial strains grown to exponential phase (optical density at 600 nm [OD_600nm_] of 0.4) were diluted in PBS to obtain 10^3^ bacteria in 10 µL and subsequently incubated with rabbit serum for 30 min at 37 °C. Next, 10^5^ PMNs put in Hank’s Balanced Salt Solution (HBSS) (Gibco, Belgium) supplemented with Mg^2+^, Ca^2+^, and Gelatin (0.1 %) was added to the bacterial solution and incubated during 45 min at 37 °C. The reaction was stopped by incubating at 4 °C for 10 min at and PMNs were lysed by adding 100 µL of sterile water. Lastly, bacteria were plated on TSB-Agar and incubated overnight at 37 °C before CFU counting.

### 2.5. Measuring the Biofilm Formation by Shigella

Two methods were used to monitor whether *Shigella* forms biofilm. First, we used the classical Crystal Violet’s (CV) binding assay [26]. Briefly, overnight cultures of bacteria were seeded in polystyrene 24-wells patches (Costar^®^) (Fisher scientific, Belgium) at 10^7^ CFU/mL in TSB medium supplemented with adequate antibiotics as needed. Bacteria were brooded for 24 h at 37 °C. Floating bacteria were removed by washing. The bacterial biofilm forming in the air–liquid interface was colored with 0.5 % CV’s solution for 20 min before washing with distilled water. Finally, the biofilm was fixed with 1 mL of absolute ethanol and was unstuck by means of a Pasteur pipette. The [OD_570 nm_] was measured and the results were expressed in percentage of the membership regarding wild-type strain.

The second method used here is based on the Biofilm Ring Test^®^ (BioFilm control, France) which is more sensitive and consisted of the immobilization of magnetic beads by attached cells as previously described [27]. Consequently, the more beads are entrapped by cells, the less they are detectable after magnetization, and subsequently, the more cells are attached to the surface. The assay was carried out in modified polystyrene 96-well microtiter plates as described in [27]. Wells containing Brain Heart Infusion (BHI) medium were inoculated with a bacterial suspension and magnetic beads and were incubated at 37 °C without shaking. Wells were scanned, magnetized, and re-scanned. Free beads were attracted to the center of the bottom of wells, forming a black spot, while beads blocked by the biofilm remained in place. As a control, BHI medium showed a centered spot. Images of each well before (I_0_) and after (I_1_) magnetization were compared with the Biofilms Control^®^ software that gives a Biofilm Index (BFI). A value of BFI > 7 indicates a high mobility of beads under magnet action (i.e., control wells) (Biofilm control, France). BFI < 2 represents a full immobilization of beads (positive biofilm), while a BFI values ranging between 2 and 7 corresponded to the course of biofilm formation. Optimal condition leading to biofilm formation was determined by growing bacteria from 1 h to 6 h.

### 2.6. Protein Purification and Maltose Binding Protein (MBP) Pull-Down Assay 

Plasmids pKA20 (MBP-SfPgdA), pKA22 (SfPgdA-His), and pKA23 (SfGtr4-His) were constructed by cloning PCR DNA fragments (856-bp *Bam*HI–*Pst*I, 1102-bp *Nco*I-*Bgl*II, and 855-bp *Nco*I-*Bgl*II) into the corresponding sites of pMALCRI or pQE60 vectors (Qiagen, France). To produce recombinants MBP-SfPgdA, SfPgdA-His, and SfGtr4-His proteins; overnight culture of *E. coli* carrying pKA20, pKA22, or pKA23 was subcultured into 100 mL of L-broth. The cultures were grown with vigorous shaking at 37 °C until an [OD_600nm_] of 0.8 was reached. Next, isopropyl β-D-1-thiogalactopyranoside (IPTG) was added at a final concentration of 0.1 mM, and growth was continued for an additional 3 h. Cells were harvested by centrifugation and stored at −80 °C until use. Cell pellets were thawed on ice, suspended in 1.5 mL of lysis buffer supplemented with Lysozyme, 1% Triton X-100 and protease inhibitor cocktail (Roche), broken by ultrasonic treatment, and then centrifuged at 6000 *g*. Next, the supernatant was retained for further protein purification steps. The maltose-fused proteins MBP-SfPgdA bound to 100 µL of amylose beads were mixed with a cleared extract of TOP10 producing recombinant SfPgdA-His or SfGtr4-His hybrid proteins and further incubated for 1 h at 4 °C. Supernatants were removed by centrifugation, and beads were washed six times with washing buffer (pH 7.4). After the final wash, supernatants were removed, and 100 µL of 10 mM of saccharose was added to elute MBP-fused protein. From each eluate, 10 to 20 µL (~100 ng) of proteins was separated by SDS-PAGE and further analyzed by Western blot using an anti-His mAb and anti-GST and anti-MBP polyclonal antibodies. 

### 2.7. Detection of the Glycosyltransferase Enzymatic Activity 

The Gtr enzymatic activity of MBP-SfGtr4 was monitored in 96-well microplates. In brief, we used an enzymatic chain reaction which allows the detection of the uridine diphosphate (UDP) product by coupling its formation to the nicotinamide adenine dinucleotide deshyrogenase (NADH) mediated reduction of pyruvate to lactate by lactic dehydrogenase. The pyruvate is produced from phospho(enol)pyruvate (PEP), which phosphorylates UDP in the presence of the pyruvate kinase. As the concentration of NADH decreased, the fluorescent signal at the emission wavelength of NADH is also decreased. The signal decrease was monitored at [OD_460 nm_] using an FLX 800 Microplate Fluorescence plate reader with excitation at 340 nm as previously described [28]. For the experimental procedure, each reaction contained buffer (50 mM 4-(2-hydroxyethyl)-1-piperazineethanesulfonic acid (HEPES), pH 7.9 (Clinisciences, France), 5 mM MgCl_2_ and, except where noted, 0.2% Triton (X-100), 0.5 mM PEP, 0.2 U/µL of lactic dehydrogenase, 3 U/µL of pyruvate kinase, 0.25 mM NADH, 15 % methanol, an appropriate amount of UDP-N-Acetylglucosamine (GlcNAc), lipid A from *E. coli*, and 0.5–10 µL of the enzyme. The total volume of each reaction was 100 µL. All the components, except for the MBP-SfGtr4 substrates and MBP used as a negative control, were premixed in a reservoir and dispensed into each well. The substrates were then added to the reaction mixtures and incubated for 5–10 min until a stable background rate was achieved. MBP-SfGtr4 was then added, and the fluorescence was monitored after 20 min.

## 3. Results

### 3.1. Purified SfGtr4 Exhibits a Glycosyltransferase Enzymatic Activity

**Figure 1:**
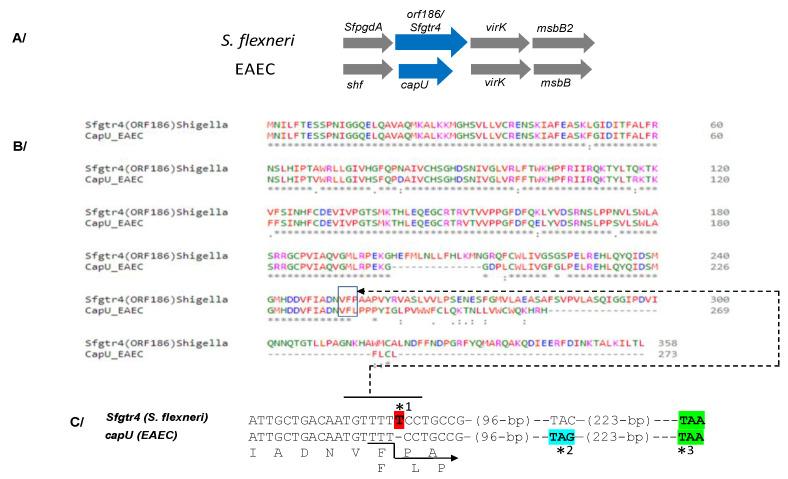
Conservation and sequence analysis between *Shigella flexneri* and *Enteroaggregative E. coli* (EAEA). (**A**) Conservation of the *sfpgdA-sfgtr4-virK-msbB2* cluster between *S. flexneri* and *Enteroaggregativ*e *Escherichia coli* (EAEC). (**B**) Sequence alignment between Sfgtr4 and CapU proteins using Clastalow version 1.2.4 (**C**) Due to the absence of thymine (T) (*1) in *capU*, the last 74 carboxyl residues of Sfgtr4 are missing in CapU. Numbers 96 and 223 correspond to fully conserved nucleotides. Arrow points to the translational shift within CapU which is prematurely stopped at codon TAG (*2). If the thymine T (*1) was conserved in *capU*, both *Sfgtr4* and *capU* would share the same stop codon (*3).

**Figure 2:**
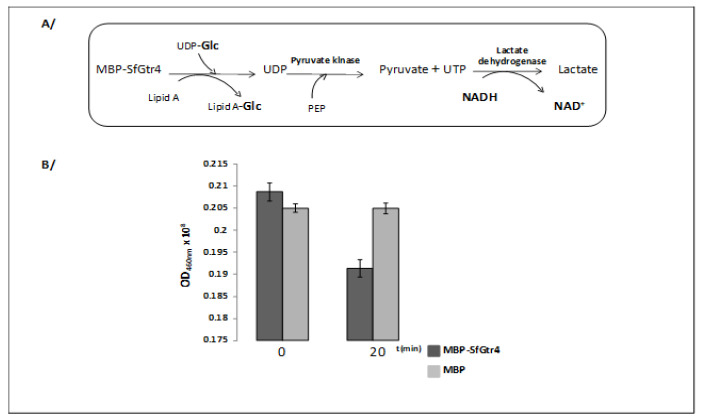
SfGtr4 exhibits a glycosyltransferase activity. (**A**) Schematic representation of the cascade of reaction used to monitor the enzymatic activity of maltose binding protein (MBP)-SfGtr4 protein. (**B**) The Gtr activity was monitored by measuring the decreased of NADH fluorescence 20 min post-incubation. Purified MBP was used as a negative control. This experiment was performed in triplicate.

The *SfpgdA-Sfgtr4*-*virK*-msbB2 genes, encoded by the virulence plasmid pWR100, were well conserved in *Enteroaggregativ*e *Escherichia coli* (EAEC) [1] (Figure 1A). Due to an in-frame mutation, sequence comparison revealed that the last 74 residues of SfGtr4 are missing from CapU (Figure 1B,C). The *Sfgtr4* gene encodes a putative Gtr related to the GT-4 family. To investigate the enzymatic activity of SfGtr4, we adapted a previously described fluorescence assay (see Material and Methods; M&M). Briefly, we used an enzymatic chain reaction which facilitates the detection of UDP product by coupling its formation to the NADH-mediated reduction of pyruvate to lactate (Figure 2A). First, we constructed plasmid pKA20 producing MBP-SfGtr4. To monitor SfGtr4’s potential glycosylation activity, 4 µg of purified recombinant MBP-SfGtr4 protein was added to the reaction mixture. As shown in Figure 2B, a 50% drop in fluorescence was detected, while no change was seen using purified MBP alone (Figure 2B). Our result demonstrates the role of SfGtr4 as a Glycosyltransferase mediating Glc transfer from UDP-Glc to lipid A (Figure 2B).

### 3.2. Inactivation of Sfgtr4 or SfpgdA Gene Reduces Shigella Entry into HeLa Cells Without Impairing Effectors Secretion via the T3SA

**Figure 3:**
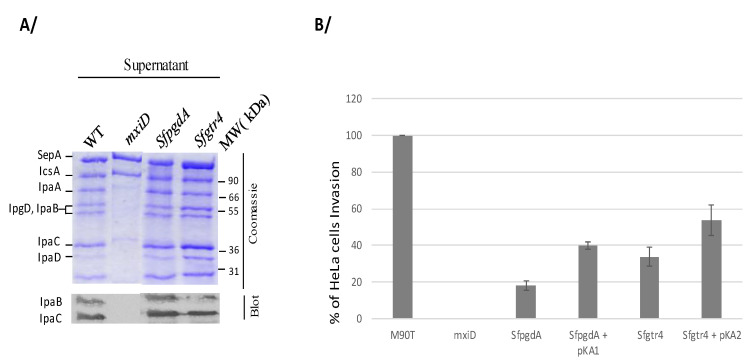
Inactivation of *Sfgtr4 or SfpgdA* impairs bacterial entry into HeLa cells without affecting secretion via T3SA. (**A**) Proteins in the supernatant were precipitated from wild-type strain (WT), *SfpgdA,* and *Sfgtr4* mutants and separated on SDS-PAGE stained with Coomassie blue or blotted using anti IpaB and IpaC mAbs. The *mxiD* mutant was used as a T3S secretion deficient control [19]. (**B**) Bacterial entry into HeLa cells was checked using the gentamicin protection assay [23]. Semi-confluent monolayer of HeLa cells was infected by WT, mutants *SfpgdA* and *Sfgtr4,* and their corresponding complemented strains with plasmid pKA1 (SfPgdA) or pKA2 (SfGtr4). Following bacterial entry, infected cells were incubated for 2 h at 37 °C and then lysed by Triton X-100. Bacterial dilutions were further plated on agar-plates, incubated at 37 °C for 16 h before CFU counting. Shown data are the mean of three independent experiments, and each one was performed in triplicate (*n* = 3).

To investigate the role of SfGtr4 in virulence, we inactivated its corresponding gene on pWR100. The mutant was first tested in vitro for effectors secretion via the Mxi-Spa T3SA. Proteins of the supernatant, obtained either under constitutive or Congo red (CR) inducible secretion condition, were separated by SDS-PAGE stained with Coomassie blue or immunoblotted using anti-IpaB and IpaC mAbs. Both *Sfgtr4* and *SfpgdA* mutants showed wild-type (WT) secretion profile (Figure 3A). As expected, no secretion was seen with the *mxiD* T3S secretion-deficient mutant, except for SepA and IcsA, the secretion of which is T3S-independent [19]. Thus, at least in vitro, both SfPgdA and SfGtr4 are not required for T3 secretion.

Secretion of effectors via the T3SA is often coupled to the capacity of *Shigella* to invade cultured cells [2]. To assess whether inactivation of *Sfgtr4* or *SfpgdA* affects bacterial entry into HeLa cells, we performed a gentamycin protection invasion assay. Compared to WT strain, the invasive capacity of the two *Sfgtr4* and *SfpgdA* mutants was reduced by 3- and 4-fold, respectively. A complementation experiment using SfpgdA/pKA1 and Sfgtr4/pKA2 strains revealed 40% and 50% WT invasion recovery, respectively (Figure 3B). This result is likely attributed to the unbalanced single SfPdgA or SfGtr4 overproduction in these strains. We conclude that both SfGtr4 and SfPgdA are prerequisite for efficient HeLa cells invasion by *Shigella*.

### 3.3. Inactivation of Sfgtr4 Gene Enhances Sensitivity to Lysozyme Action and Increases Bacterial Killing by Polymorphonuclear Neutrophils (PMNs) 

**Figure 4:**
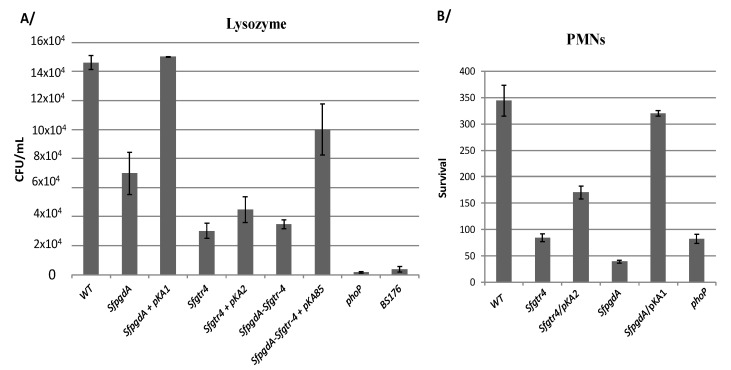
Inactivation of the *Sfgtr4* gene increases sensitivity to lysozyme and enhances bacterial killing by polymorphonuclears neutrophils (PMNs). (**A**) Bacteria grown to exponential phase were incubated for 4 h at 37 °C with lysozyme from chicken egg (40 µg mL^−1^) supplemented with lactoferrin (0.1 µg mL^−1^). 10^3^ bacteria were incubated for 30 min with serum of rabbit at 37 °C and further incubated for 45 min with PMNs extracted from human blood. (**B**) PMNs were lysed using distillated water before CFU counting after 24 h of incubation at 37 °C. Strains used are: WT, mutants: *SfpgdA, Sfgtr4*, *SfpgdA-Sfgtr4* (double mutant), and *phoP.* Plasmids pKA1 (SfPgdA), pKA2 (SfGtr4), and pKA85 (SfPgdA and StGtr4) were used in the complementation experiments. Shown data are the mean of three independent experiments and each one was performed in triplicate (*n* = 3).

We have previously shown that inactivation of *SfpdgA* rendered *Shigella* more sensitive to lysozyme action [12]. A similar phenotype was obtained here with the *Sfgtr4* mutant, roughly rescued upon complementation with plasmid pKA2 (SfGtr4) (Figure 4A). As a control, we used the *SfpgdA* mutant, which exhibited less pronounced sensitivity compared to the *Sfgtr4* mutated strain (Figure 4A). Next, we tested whether simultaneous mutation of the two studied genes would strongly affect sensitivity to lysozyme action. For that, we constructed a *Shigella* strain lacking both *SfpgdA* and *Sfgtr4* genes and found that the latter exhibited the *Sfgtr4* mutant’s sensitivity to lysozyme action (Figure 4A). The parental phenotype was partially restored (~70%) upon complementation with plasmid pKA85 producing both SfPgdA and SfGtr4 (Figure 4A). This result indicates the lack of a synergetic effect in the double mutant. 

It is well established that among bactericidal mechanisms elaborated by PMNs, some require fusion of bacteria in phagosomes with granules that contain high concentrations of lysozyme and lactoferrin [24]. Thus, we investigated the behavior of the *Sfgtr4* mutant upon PMNs infection. As for the *SfpgdA* and *phoP* mutants [12], used here as a control, inactivation of *Sfgtr4* reduced bacterial persistence within PMNs, a phenotype that was partially rescued (~50%) upon complementation with plasmid pKA2 (Figure 4B). In contrast, compared to the *Sfgtr4* mutant, survival of the *SfpgdA* mutant was drastically affected, while strain *SfpgdA*/pKA1 recovered almost the parental phenotype. SfGtr4 is therefore required for *Shigella* survival within PMNs.

### 3.4. Inactivation of Sfgtr4 or SfpgdA Accelerates Biofilm Appearance

**Figure 5:**
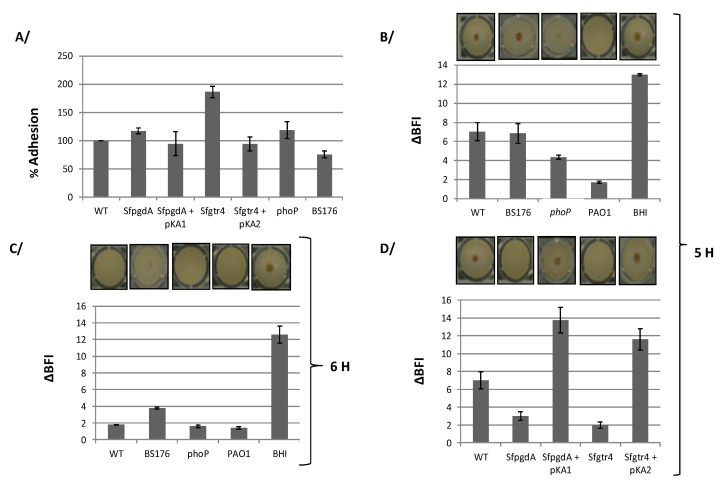
*S. flexneri* forms biofilm and deletion of either *SfpgdA* or *Sfgtr4* accelerates this formation. (**A**) Quantification of biofilm formation by crystal violet (CV) assay. Bacteria were grown in tryptic casein soy broth (TSB) medium during 24 h at 37 °C and Biofilm formation was monitored by measuring the absorbance at 570 nm. Strains used are WT, mutants *SfpgdA*, *Sfgtr4, phoP*, and BS176 (pWR100 cured *S. flexneri* strain). Biofilm formation was also measured using the biofilm ring test after bacterial incubation at 37 °C during 5 h (**B**,**D**) and 6 h (**C**). ΔBFI (Biofilm Formation Index) represents (BFI controls−BFI samples). Plasmids pKA1(SfPgdA) and pKA2 (SfGtr4) were used in the complementation experiments. Wild-type *P. aeruginosa* (PAO1) and BHI medium were used as positive and negative controls, respectively. Shown data are the mean of three independent experiments, and each one was performed in triplicate (*n* = 3).

Both SfGtr4 and SfPgdA are related to bacterial proteins required for biofilm formation, a process that remains poorly studied in *Shigella*. We first tested the capacity of *S. flexneri* strains to adhere on polystyrene plastic surfaces using the CV binding assay (see M&M). Although WT *Shigella* does not efficiently adhere to this type of support compared to the strong adhesion seen with the *Pseudomonas aeruginosa* PAO1 strain, we found that WT strain, grown for 24 h at 37 °C, formed biofilm (Figure 5A). Comparatively, the *Sfgtr4* mutant exhibited an increased bacterial adhesion (~80%), a phenotype that was almost restored to the WT level in strain *Sfgtr4/*pKA2 (Figure 5A). In contrast, only a limited increase in adhesion (~17%) was seen in the *SfpgdA* mutant (Figure 5A). All observed phenotypes were not associated with differential bacterial growth since the growth rate of either WT, *SfpgdA,* or *Sfgtr4* mutant was equivalent (data not shown). Compared to WT *Shigella*, *Pseudomonas aeruginosa* (PAO1) strain exhibited 2.4-fold more abundant biofilm (data not shown).

To gain further evidence in support of the biofilm formation, we next use the ring sensitive assay (M&M). Wild-type *Shigella* was grown at 37 °C under various incubation times to determine condition during which a centered spot at the bottom of the well would disappear (M&M). Up to 5 h of growth, the spot was still detectible (Figure 5B). Interestingly, after 6 h growth, no spot was seen, suggesting biofilm formation by WT *Shigella* was completed (Figure 5C). As a control, biofilm formation in strain PAO1 occurred within 2 h of growth (Figure 5B,C). As a confirmation these beads were not blocked by other factors other than sessile cells, BHI medium showed a centered spot (Figure 5B,C).

Having optimized the condition under which WT *Shigella* developed biofilm, we next explored the behavior of *Sfgtr4 and SfpgdA* mutants in this assay. Remarkably, no spot was seen with these strains after 5 h growth (Figure 5D). Interestingly, following 5 h growth, strains *Sfgtr4/*pKA2 and *SfpgdA/*pKA1 recovered the parental phenotype (Figure 5D).

### 3.5. The phoP Mutant, but not the pWR100 Shigella Cured Strain, Increases Biofilm Formation

PhoP regulates the transcription of *SfpgdA-Sfgtr4-virK-msbB2* cluster, and SfPgdA protein is not produced by the *phoP* mutant [12]. Based on this, we investigated biofilm formation by the *phoP* mutant. In the CV binding assay, compared to WT strain, a relative increase in adhesion (~18%) was detected with the *phoP* mutant (Figure 5A). Furthermore, using the ring test, after 5 h growth, biofilm was seen with the *phoP* mutant, in a comparable manner to the *SfpgdA* or *Sfgtr4* one (Figure 5B).

As the two studied genes are encoded by pWR100, we next carried out similar experiments using BS176, a pWR100 cured *Shigella* strain. In the CV binding assay, BS176 exhibited a ~25 % decreased adhesion compared to WT strain (Figure 5A). In the ring test, post 5 h growth, a spot was observed in the center of the well (Figure 5B). When tested after 6 h growth, the intensity of the spot was slightly reduced (Figure 5C). This result is likely to be associated with the fast growth of BS176 compared to WT strain (data not shown). We conclude that BS176 does not form biofilm, which strongly suggests the need for additional pWR100 encoding factors.

### 3.6. SfGtr4 Interacts with SfPgdA

**Figure 6:**
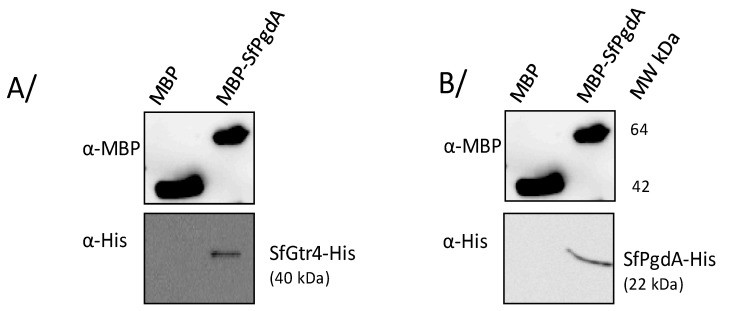
SfPgdA interacts with itself and with SfGtr4 using maltose binding protein (MBP) pull-down assay. MBP-SfPgdA bound to amylose beads was mixed with a cleared extract of *E. coli* strain producing either (**A**) recombinant His-SfGtr4 or (**B**) His-SfPgdA hybrid proteins. Saccharose was used to elute MBP-fused proteins. Aliquots of each eluted fraction were analyzed by Western blot using an anti-His mAb or anti-MBP polyclonal antibodies. Purified MBP alone was used as a negative binding control. MW: proteins molecular weight.

The contiguous localization of *SfpgdA* and *Sfgtr4* genes was associated with similar phenotype shared by their respective mutants: (i) reduced entry into HeLa cells; (ii) increased biofilm formation; and (iii) increased susceptibility to lysosomal degradation and killing by PMN. Such outcomes prompted us to investigate the potential interaction between their products. We therefore constructed two plasmids producing SfPgdA-His and SfGtr4-His and used another available one producing maltose binding protein fused to SfPgdA (MBP-SfPgdA) [12] (Table 1). Using the MBP pull-down assay (M&M), we demonstrated that SfPgdA self-associates and interacts with SfGtr4 (Figure 6). As a control, no interaction was detected with MBP alone.

## 4. Discussion

The present study highlights the ability of bacterium of the genus *Shigella* to form biofilm. We report here that the deletion of either *Sfgtr4* or *SfpgdA* genes accelerates biofilm appearance. Both SfGtr4 and SfPgdA are closely related to Shf and CapU proteins required for *Enteroaggregative E. coli* (EAEC) biofilm formation [3,5,15]. In the latter, strong aggregation in culture medium causes the spontaneous settling of cells onto the substratum and subsequently contributes to thick biofilm formation [3]. It was suggested that *shf* gene is not related to aggregation in the liquid phase and adhesion in the early phase but is required to form the multiple layers of biofilm in the maturation phase [3,26]. In addition, the same authors showed that strain mutated in *capU* gene exhibited parental EAEC biofilm, while our inactivation of *the Sfgtr4* gene rather accelerated its appearance in *Shigella*. The difference between our data and those of Fujiyama et al. [3,26] in EAEC could be due to the lack, within CapU, of the 74 carboxyl-terminal residues of SfGtr4 (Figure 1B). Thus, production of SfGtr4 by WT *Shigella* prevents biofilm appearance, while its deletion rather mimics both WT EAEC and *capU* mutant phenotype.

Biofilm formation comprises different steps; one of the earliest is the attachment of the bacteria on the substrate. The absence of a biofilm formation in BS176 supports the existence of factors that are encoded by the pWR100 plasmid that are paramount in biofilm formation. In support of this, we demonstrated a molecular interaction between SfPgdA and SfGtr4. We have previously demonstrated that SfPgdA is associated to the bacterial membranes fraction [12]. It is likely that both proteins associate in a complex dealing with bacterial surface modifications. Comparatively, PGA production and excretion changes at the bacterial envelope were suggested to be associated with the PgaABCD complex of *E. coli* [5]. Biofilm is generally embedded in extracellular polysaccharide composed of PGA [29], and its formation is mediated by exopolysaccharide (EPS) production [30].

SfPgdA exhibits a peptidoglycan deacetylase (PGD) activity used by WT *Shigella* to resist lysozome hydrolase activity [12]. Considering that PG breaks, generated by PG hydrolase and facilitate biofilm formation [31], *Shigella* strain lacking SfPgdA becomes more sensitive to hydrolase, which in turn accelerates biofilm formation. Evidence supporting this finding was reported in *Lactococcus lactis* whose biofilm formation was mainly attributed to PG hydrolysis [30]. Moreover, PGA deacetylation in *Streptococcus epidermidis* contributes not only to biofilm development but also to immune evasion and virulence [18], a result that corroborates the recent role ascribed to SfPgdA in *Shigella* persistence within PMNs [12].

The virulence processes of pathogenic bacteria are frequently coupled to the secretion of effector molecules into the environment. T3SA is required for protein secretion that contributes to the virulence of several animal and plant pathogens [2,32,33] and is responsible for *Shigella* entry into epithelial cells [8,18]. One unexpected result, obtained here, points to the reduced invasion of HeLa cells by *SfpgdA* and *Sfgtr4* mutants, although both strains exhibit a functional T3SA. A similar phenotype was previously reported with the *gtrV* gene mutant of *Shigella* [34]. Interestingly, GtrV is involved in LPS glucosylation [34]. The invasion defect of the *gtrV* mutant was attributed to the increased length of the unglugosylated LPS, which consequently extend both the glucosylated one and the T3S needle 50 nm length entities [34]. One may consider that the lack of the Gtr enzymatic activity in the *Sfgtr4* mutant could disrupt needle exposition at the bacterial surface, which consequently reduces the capacity to invade HeLa cells. Our invasion data revealed partial complementation using either *sfgtr4/*pKA2 or *sfpgdA*/pKA1 strains. Both pKA1 and pKA2 plasmids are derivative of the high copy number pUC18 vector known to constitutively overproduces proteins. As SfPgdA is associated with the bacterial membrane fraction [12] and interacts with SfGtr4, their single overproduction could significantly affect their function. Interestingly, in the lysozyme assay, simultaneous overproduction of both SfPgdA and SfGtr4, in the double *SfpdgA-Sfgtr4* mutant, recovered almost 70% of the WT phenotype. A finding that contrasts with the lowest complementation seen following individual overproduction of either SfpgdA or Sfgtr4. Such a finding suggests that the identified molecular interaction between SfPgdA and SfGtr4 is likely involved in the regulation of their functions.

How does *Sfgtr4 mutant* affect *Shigella’s* resistance to lysozyme action? It is well documented that LPS glycosylation destabilizes the bacterial membrane, which facilitates lysozyme access to the PG layer [7]. For example, inactivation of *Salmonella enterica ugtl* gene, involved in heptose incorporation into lipid A, increased sensitivity to polymyxin B [18]. Interestingly, the *SfpgdA-msbB2* locus and *ugtl* gene are both regulated by the PhoP/PhoQ regulatory system [7,10]. Since PhoP is involved in the regulation of genes remodeling bacterial cell envelope in response to changing environmental conditions, the increased biofilm formation detected in the *phoP* mutant is likely to be attributable at least to the lack of SfPgdA [12].

Based on our reconstituted in vitro Gtr enzymatic assay, we cannot precisely statute about Sfgtr4’s target—either LPS or PG, or both? By transferring glucose to lipid I, SfGtr4 may glycosylate PG. Comparatively, MurG, a Gtr from *E. coli*, transfers a molecule of glucose to lipid I, which in turn generates lipid II, the PG precursor [28]. The lack of PG glycosylation in the *Sfgtr4* mutant would amplify lysozyme action as previously reported for the *namH* mutant of *Mycobacterium smegmatis* [35].

To date, no investigation has identified the environmental ecology niches of *Shigella* yet. Here, we bring an additional contribution on biofilm formation by WT *Shigella,* a process that we showed here to be controlled by SfPgdA and SfGtr4 proteins. A previous study reported that exposition of WT *Shigella flexneri* to bile salts induced biofilm formation and revealed the role of LPS O-antigen synthesis in this process [36]. Indeed, various rough mutants or mutations in the *galU* gene, which catalyzes the formation of UDP-glucose, required for O-antigen synthesis, affect bile salts resistance. Another study reported that a *Shigella* strain lacking the *icsA* gene, required for both adherence to cells and bacterial actin-based motility, abolished bile salt-induced biofilm formation [37]. Here, we showed that production and secretion of IcsA were not affected in the *SfpgdA* and *Sfgtr4* mutants (Figure 3A), suggesting the existence of additional mechanisms involved in biofilm formation by *Shigella*. More recent study reported that the biofilm formation by Gram-negative *Haemophilus influenzae* was increased by deletion of *galE* gene and the remarkable availability of UDP-GlcNAc precursors [38]. The same study reported that the suppression of alternative lipooligosaccharides glycosyltransferase activity by UDP-Galactose epimerase enhanced murine lung infection and evasion of serum IgM [38].

The arising question here is why and when *Shigella* would need to form biofilm. If we consider that only 10 to 100 bacteria are sufficient to cause disease in humans, such bacterial dose cannot bind to a biofilm. Nevertheless, one could suggest that biofilm development is a transitory regulated event that occurres upon food contamination to help *Shigella* survival outside of its preferred habitat. This phenomenon may happen under environmental conditions during which the expression of a limited number of genes, including *SfpgdA* and *Sfpgtr4,* is downregulated.

Compared to macrophages, neutrophils prevent the escape of *Shigella* from phagocytic vacuoles in which the bacteria are killed [39]. The *Sfgtr4* and *SfpgdA* mutants are both unable to escape PMNs killing. One might consider that the ability of WT *Shigella* to modify its surface, by targeting either PG and/or LPS, is to subvert bacterial detection by the host immune system. Neutrophil-phagocytosed *Shigella* are killed rapidly by different mechanisms including proteolytic enzymes, antimicrobial proteins, and reactive oxygen species [38]. Thus, further investigations are needed to unravel mechanisms underpinning *Shigella*’s circumvention and/or counteraction of the host’s immune responses.

## Figures and Tables

**Table 1:** Plasmids and strains used in this study (TS)

**Plasmid**	**Characteristics**	**Reference**
pKA1	pTZ18R encoding native SfPgdA	[12]
pKA2	pTZ18R encoding native SfGtr4	TS
pKA85	pTZ18R encoding native SfPgdA and SfGtr4	TS
pKA4	pSW23T- *SfpgdA::aphA-3* (Suicide vector)	[12]
pKA8	pSW23T- *Sfgtr4::aphA-3* (Suicide vector)	TS
pKA121	pSW23T- *SfPgdA -Sfgtr4::aphA-3* (Suicide vector)	TS
pKA20	pMALCRI expressing MBP-SfPgdA	TS
pKA22	pQE60 expressing SfPgdA-His6	TS
pKA23	pQE60 expressing SfpgDA-His6	TS
**Strain**	**Genotype**	**Reference**
M90T-Sm	Derivative of wild-type *Shigella flexneri* strain M90T	[16]
BS176	M90T-Sm pWR100 cured strain	[18]
SF401	M90T-Sm *mxiD* (*mxiD::aphA-3*)	[19]
SBkr1	M90T-Sm *SfpgdA* (*SfpgdA::aphA-3*)	[12]
SBkr2	M90T-Sm *Sfgtr4* (*Sfgtr4::aphA-3*)	TS
SBkr3	M90T-Sm Sf*pgdA* Sf*gtr4* (*SfpgdA-Sfgtr4::aphA-3*)	TS
PhoP	M90T-Sm *phoP* (*phoP::aphA-3*)	[12]

**Table 2:** List of primers used in this study.

Name	Primers 5′–3′	R.S
sfpgdA.1	CGGGATCCTTTAAACGAAGGGGGCATTTTG *	*Bam*HI
sfgtr4.1	CGGGATCCGGAAAGTTGCGCATGGCTG	*Bam*HI
sfgtr4.2	ACGCGTCGACCCGATAAATGATAAGTTACTTAC	*Sal*I
sfgtr4his-s *	GTCCATGGGATTAAACGAAGGGGGCATTTT	*Nco*I
sfgtr4his-as **	GCTAGATCTCATCCGGTAATCTTGGCCCC	*Bgl*II
sfpgdAmal-s	CGGGATCCTTAAACGAAGGGGGCATT	*Bam*HI
sfpgdAmal-as	AACTGCAGTTAATCATCCGGTAATCTTGGC	*Pst*I
sfpgdAhis-s	GTCCATGGGAAATATACTATTTACGGAATCAT	*Nco*I
sfpgdAhis-as	GCTAGATCTCTTGTGCTTCGCTAATGTGAG	*Bgl*II

R.S: Engineered restriction sites are underlined. * s: sense and ** as: antisense.
